# The Relationship of Parasite Allergens to Allergic Diseases

**DOI:** 10.1007/s11882-023-01089-8

**Published:** 2023-06-03

**Authors:** Luis Caraballo, Kevin Llinás-Caballero

**Affiliations:** Institute for Immunological Research, University of Cartagena, Cartagena de Indias, Colombia

**Keywords:** Helminth, Asthma, Allergy, House dust mites, *Ascaris*, IgE

## Abstract

**Purpose of Review:**

Helminth infections modify the natural history of allergic diseases, by either decreasing or increasing their symptoms. Several helminth components are involved in the increasing of the allergic response and symptoms, overcoming the concomitant immunosuppression of helminthiases. However, the role of individual IgE-binding molecules in this process remains to be defined.

**Recent Findings:**

We updated the list of helminth allergens and IgE-binding molecules, their effects on asthma presentation, and their impact on allergy diagnosis. Data from genetic and epigenetic studies of ascariasis are analyzed. A new species-specific *A. lumbricoides* allergen has been discovered, with potential use in molecular diagnosis.

**Summary:**

Most helminth IgE-binding components are not officially classified as allergens in the WHO/IUIS database, although there is evidence of their influence increasing allergic manifestations. Further immunological characterization of these components is needed to better understand their mechanisms of action and evaluate the ways in which they can influence the diagnosis of allergy.

## Introduction

Most parasite allergens come from helminths, including those causing soil-transmitted infections (*Ascaris lumbricoides*, *Strongyloides stercoralis*, *Trichuris trichiura*, and the hookworms *Necator americanus* and *Ancylostoma duodenalis*), filarial nematodes (*Wuchereria bancrofti*, *Brugia malayi*, *Onchocerca volvulus*), and platyhelminth flukes (*Schistosoma haematobium*,* S. mansoni*,* S. japonicum*). During infection, helminths produce a variety of molecules for their metabolism and survival as parasites, some of them stimulate the synthesis of specific IgE, and a small number induce allergic symptoms and/or increase the frequency and severity of allergic diseases. The clinical effects of this pro-allergenic activity depend on the modulation of host immunity exerted by the parasites, which means that they must overcome the concomitant immunosuppression to be apparent.

The degree of immunosuppression varies with the type of parasite and host genetic background; in addition, it has been associated with the severity of infection, but the reasons why some people develop severe infections, while others do not, remain to be defined. The contemporary version of the hygiene hypothesis proposes that, among other factors, the reduction of helminth infections across the world (i.e., reduction of immunomodulation) has led to an increase in allergic diseases [1]. Therefore, the potentiating effect of helminthiases on allergic responses is just an aspect of the complex host-parasite interactions between humans and those organisms. Studies of these interactions have been fundamental for understanding basic aspects of both allergy and parasitology; in addition, they have unveiled interesting clinical repercussions that deserve consideration in our medical practice. In this review, we describe the role of some helminthiases and helminth allergens as potentiators of the allergic response, mainly in the tropics, where they are endemic. We will also discuss the impact of helminth immunity on the diagnosis of allergic diseases such as asthma. A detailed review of *Anisakis* spp. allergens can be found elsewhere [2] and will not be included in this review.

## Helminthiases and Allergic Diseases

The first observed allergy-like manifestations induced by helminths were symptoms of respiratory distress or hives in children suffering *A. lumbricoides* infection (ascariasis); however, these and other clinical manifestations also occur during other helminthiases. These infection-induced symptoms are present in a minority of the population, probably because it is genetically predisposed to overreact to parasitic infection, and the degree of concomitant immunosuppression is not sufficient to reduce the manifestations of allergy, especially if the infection is acute and mild. Numerous studies have evaluated the pro-allergenic effects of helminthiases on allergic diseases, and a recent meta-analysis concluded that helminth infections may increase the risk of bronchial hyperreactivity in children and atopy in adults; most of these effects associated with *A. lumbricoides* infection [3••]. However, the role of individual helminth allergens as potentiators of the allergic response has been little explored.

**Fig. 1:**
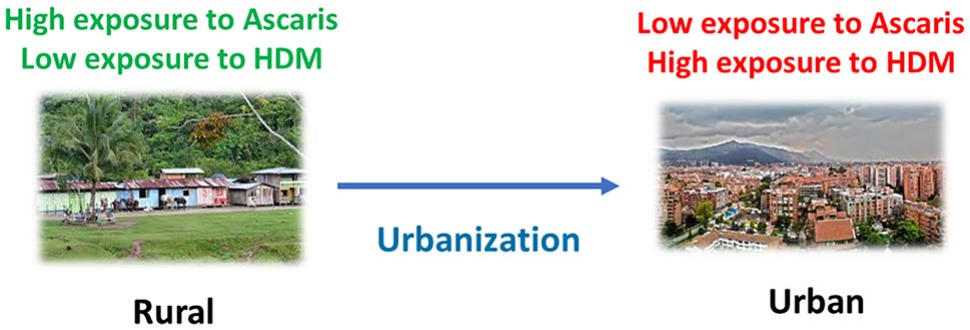
Urbanization and allergies in the tropics: the influence of helminthiases and house dust mites. If we look at the current situation in the tropics, we can hypothesize that one of the reasons why urbanization leads to an increase in allergy is the reduction of severe forms of helminthiases due to the improvement of hygiene conditions associated with urbanization

Helminthiases have been controlled in most developed countries but are still endemic in tropical regions; for example, ascariasis has decreased during the last 60 years in industrialized countries, and currently, the most severe infections are present in some rural tropical villages. In tropical urban areas, hygienic conditions are better and regular deworming campaigns are carried out, which diminish the frequency and intensity of infections. Therefore, the effects of helminth allergens on allergic diseases are mostly observed in urban zones of developing countries, although they could also be present in some temperate regions [4•]. Urbanization in developing countries has had several effects [5•], such as providing better conditions for house dust mite (HDM) proliferation and better hygiene conditions associated with less helminth exposure, which supports the high HDM sensitization and allergy (Fig. 1). Moreover, current trends of climate change are expected to expand the geographical extent of HDM growth and sensitization [6].

## Helminth Allergens

Helminth allergens are the result of normal type 2/IgE immune responses during infections. Animal models have shown that specific IgE antibodies, together with eosinophils, can destroy helminth larvae, and other type 2 immune mechanisms have been revealed using diverse helminth species in animal models of infection [7, 2], but their clinical relevance in terms of modifying allergic diseases deserves more investigation. All these molecules are allergenic (they induce and bind IgE), but only few have been investigated for allergenic activity (the capacity to induce allergic inflammation), which is a crucial step in defining their clinical relevance [8]. This consideration is, of course, valid for common allergens but is particularly important for helminths because IgE-binding molecules such as *A. lumbricoides* cystatin (Al-CPI) do not have relevant allergenic activity but strong immunomodulatory effects [9]. In this sense, Table 1 shows the immunological characterization of some helminth IgE-binding molecules.

**Table 1:** Allergenic activity assays of some helminth IgE-binding molecules

**Helminth**	**Molecule**	**In vivo provocation tests**	**In vitro provocation tests**	**Animal models**	**Association with allergic diseases**
***A. suum***	ABA-1 / Asc s 1	n/d	HR	n/d	n/d
***A. lumbricoides***	Asc l 3	ST	HR	n/d	Asthma
	Asc l 5	n/d	BAT	sIg, PCA	n/d
	Asc l 13	ST	n/d	n/d	n/d
***S. stercoralis***	NIE	n/d	HR	n/d	n/d
***S. mansoni***	SmTAL1	n/d	RBL	n/d	n/d
	SmTAL2	n/d	RBL	n/d	n/d
	SmVAL4	n/d	n/d	sIg, AAI, PCA	n/d
	SmVAL26	n/d	n/d	sIg	n/d
	IPSE/alpha-1	n/d	RBL	n/d	n/d
	SmATPDase2	n/d	n/d	sIg	n/d
	SmCB1	n/d	n/d	sIg	n/d
***B. malayi***	TTR	n/d	n/d	PA	n/d
	Bm23-25 (γ-GT)	n/d	n/d	sIg	TPE
	BmAl-1	n/d	n/d	sIg	n/d
***O. volvulus***	OvTrop	n/d	HR	n/d	n/d
***N. americanus***	Calreticulin	n/d	HR	n/d	n/d
	Na-ASP-2	ST	n/d	n/d	Urticaria
***A. caninum***	Ac68	n/d	n/d	n/d	Eosinophilic enteritis

*AAI* allergic airway inflammation, *BAT* basophil activation test, *HR* histamine release, *PA* passive anaphylaxis, *PCA* passive cutaneous anaphylaxis, *RBL* rat basophil leukemia cells test, *sIg* specific immunoglobulin (IgG1 and/or IgE) response, *ST* skin test, *n/d* no data

### *Ascaris lumbricoides*

During larval pulmonary transit, *A. lumbricoides* induces respiratory symptoms similar to asthma, and sometimes Löffler’s syndrome. Also, there is epidemiologic evidence that ascariasis increases the frequency and symptoms of asthma. This potentiating effect can be detected at the population level, as it has been repeatedly observed that sensitization to *A. lumbricoides* is associated with a higher prevalence of asthma [10, 11] and asthma severity [4, 12, 13], especially in urban areas with lower exposure and mild infections. These changes are also detected at the cellular and molecular levels, with an increase in several components of the type 2 response, particularly the production of specific and cross-reactive IgE against parasite antigens [11].

**Fig. 2:**
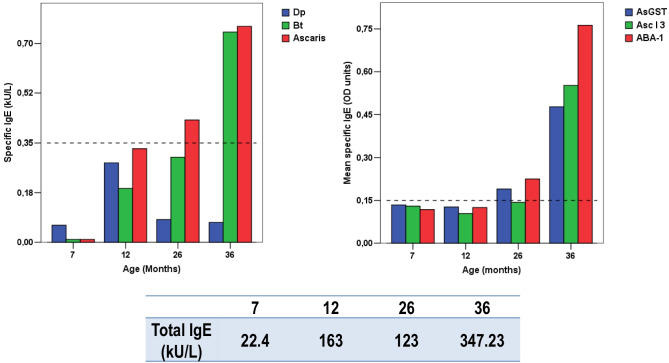
An example of allergic sensitization in a child living in the tropics. Specific IgE against *D. pteronyssinus* (Dp), *B. tropicalis* (Bt), and *Ascaris* extracts were measured using ImmunoCAP™ 100. Specific IgE to *A. lumbricoides* GST (AsGST), tropomyosin (Asc l 3), and ABA-1 were measured by ELISA. Samples were collected at 7, 12, 26, and 36 months. Data on the child’s total IgE levels at these time points are also included. Figure obtained from data of the FRAAT cohort (reference 16)

Specific IgE to HDM is the most important risk factor for asthma in the tropics. Thus, any condition that increases the allergic response against them could also increase the symptoms and severity of this disease. Since HDM exposure is high and permanent in tropical environments and induces IgE sensitization at a very early age (Fig. 2), the cross-reactivity between several allergens from both sources (e.g., tropomyosins, glutathione S-transferases [GSTs], and other non-characterized components) may explain this potentiating effect of ascariasis [10]. In addition, ascariasis might induce polyclonal nonspecific stimulation of B cells [14]; therefore, it is possible that the implicated components also stimulate HDM allergen-specific memory B cells that are permanently stimulated in the tropics. Furthermore, experimental work suggests that ascariasis can boost IgE/Th2 responses to bystander antigens [15]. These data suggest that helminthiases can boost the allergic responses to HDM in several ways.

Trying to further explore this boosting effect in human ascariasis, we studied the evolution of the immune responses in children of the FRAAT birth cohort [16], finding that *Ascaris*-sensitized children had stronger IgE to *B. tropicalis* and those sensitized to the *Ascaris* allergen ABA-1 had increased IgE responses to Blo t 5 and Blo t 12, two species-specific allergens of *B. tropicalis*, ruling out the effect of cross-reactivity [17]. This nonspecific boosting was also detected in a large population co-exposed to *A. lumbricoides* and HDM, where subjects sensitized to ABA-1 (a marker of ascariasis) had at least twice the odds of being sensitized to HDM [10]. Furthermore, in another cohort of asthmatic patients, *Ascaris*-sensitized patients had significantly higher IgE levels to the HDM-specific allergens Der p 2 and Blo t 5 [13]. These findings support the idea that the Th2/IgE hyperresponsiveness induced by *A. lumbricoides* infection includes responses not only to helminth antigens but also to HDM allergens. However, the role of *A. lumbricoides* components, the host genetic background, and the epigenetic modifications that influence this boosting effect are only beginning to be unraveled.

In a search for genetic variants supporting the IgE hyperresponsiveness of ascariasis, we found that SNPs of the genes coding for acidic mammalian chitinase (*CHIA*) and chitinase 3 like 1 (*CHI3L1*) were significantly enriched in high IgE responders to the resistance marker ABA-1; in addition, SNPs in the tumor necrosis factor superfamily member 13b gene (*TNFSF13B*) encoding the cytokine B cell activating factor were associated with high levels of total IgE [18]. We then investigated the histone 4 acetylation (H4Ac) of these and other immune genes in *A. lumbricoides*–infected and non-infected subjects, finding significant associations between H4Ac levels in the *IL4* and *CHI3L1* genes and IgE levels to *A. lumbricoides.* In addition, the levels of specific IgE antibodies to HDM were associated with H4Ac levels in the *TNFSF13B* gene. These results suggest that acariasis induces histone acetylation of immune response genes, which in turn increases the IgE response to both helminth and HDM allergens [19•]. Therefore, regarding the boosting effects of ascariasis on the allergic response, we hypothesized that patients predisposed to asthma, with a strong pro-Th2 genetic background, parasitized at an early age, under regular deworming campaigns (followed by re-infections), and permanently exposed to mite allergens, have a stronger IgE response to allergens and more severe asthma clinical symptoms.

Immunochemistry analyses of *A. lumbricoides* extract suggest that it has high cross-reactivity with *B. tropicalis* and *D. pteronyssinus*, and that this nematode has at least nine IgE-binding components [20]; however, only three have been characterized and listed in the official WHO/IUIS site. Two of these, Asc l 3 (tropomyosin) and Asc l 13 (GST), cross-react with their HDM group 10 and group 8 homologs [21, 22]. More recently, we discovered a third *A. lumbricoides* allergen, Asc l 5; the evaluation of its allergenic activity included determination of IgE-binding frequency (in two populations: 254 children and 298 all-age subjects), CD203c-based basophil activation tests, and a passive cutaneous anaphylaxis mouse model [23••]. Further analyses of this allergen, employing immunoblotting and mass spectrometry of *A. lumbricoides*, *B. tropicalis*, and *D. pteronyssinus* extracts, suggest that it is *A. lumbricoides* species-specific [24].

Tropomyosin, a well-known pan-allergen, is involved in *Ascaris*-HDM cross-reactivity [20, 25]. Specific IgE levels to Asc l 3 are significantly higher in asthmatic patients than in healthy controls, which suggests that it may be a risk factor for asthma symptoms in the tropics [10], as it is the only helminth allergen directly associated with an allergic disease. More detailed information about this pan-allergen can be found in reference [26]. Although the allergenic activity of *Ascaris* GST (Asc l 13) has not been properly defined, its recombinant form induces skin wheal and flare reaction in subjects with ascariasis. The frequency of IgE reactivity to this molecule is low (< 20%) but may be clinically relevant in cases where there is also sensitization to mite and cockroach GSTs [22]. The allergenic cross-reactivity between GSTs from *Ascaris* and other allergenic sources is supported by the structural homology between Asc l 13 and allergenic GSTs in cockroaches (Bla g 5) and HDM (Der p 8 and Blo t 8) [27]. A comprehensive review of GST allergens from different sources has been published elsewhere [28•]. The first allergen to be reported from *Ascaris* spp. was ABA-1 (Asc s 1), discovered in *Ascaris suum.* It is a polyprotein abundant in the pseudocelomic fluid of both *A. suum* and *A. lumbricoides*. In humans, IgE and IgG responses to ABA-1 have been associated with protection [29] rather than allergy symptoms. In addition, since it has no cross-reactivity with any HDM component, it is considered a marker for *Ascaris* infection.

### *Strongyloides stercoralis*

Strongyloidiasis can be asymptomatic [30], but in some cases can elicit allergic respiratory or cutaneous manifestations. Murine models show that this parasite induces airway hyperreactivity with asthma-like characteristics, such as eosinophilic inflammation, mucus hypersecretion, and bronchial wall thickening [31]. In humans, the infection can manifest with chronic cough, mimic asthma exacerbations, or worsen stable asthma, thus complicating the evolution and management of this disease [32, 33•, 34]. Localized skin eruption (i.e., larva currens) and urticaria are among the cutaneous manifestations of strongyloidiasis [35]. However, the individual components that induce these allergic symptoms or changes in the natural history of allergic diseases remain unknown.

Immunity to *S. stercoralis* involves type-2 pathways, including IgE antibodies. Both somatic antigens and excretory/secretory products (ESPs) are recognized by IgE, trigger IgE-dependent histamine release from basophils, and induce positive skin test reactions [36]. However, few individual IgE-binding molecules have been studied. For instance, NIE is a larval component recognized by human IgE that induces histamine release from basophils in 90% of patients with strongyloidiasis [37], and shares a cross-reacting C-terminal epitope with the Hymenoptera venom allergens Ves v 5 and Pol a 5 [37, 38]. Strongylastacin is another excretory/secretory metalloprotease with IgE reactivity that helps larvae penetrate the skin [39]. Anti-strongylastacin IgE was present in 93% of infected subjects but not in healthy or *Wuchereria bancrofti*–infected individuals [39]. Finally, the *S. stercoralis* IgE-binding recombinant protein rA133 is recognized by IgE from 100% of infected patients but not from healthy controls or individuals infected with other parasites [40].

### *Schistosoma *spp

Schistosomiasis has been regarded as a protective factor against atopy and asthma [41, 42], depending on the parasite burden and chronicity of the infection [43]. However, some studies have shown that anti-schistosome IgE is positively associated with specific sensitization (e.g., to mites or cockroaches) and allergic diseases [3, 44, 45]. In addition, in vivo models have used sensitization and challenge with *Schistosoma* eggs to induce type-2 inflammation [46], suggesting the presence of components with allergenic activity.

Some *Schistosoma* components are recognized by IgE, including a 22.6-kDa molecule from *S. mansoni* (Sm22.6, currently known as SmTAL1) that is associated with human resistance to reinfection [47], and the antigens Sj22.6 and Sh22.6 from *S. japonicum* and *S. haematobium* respectively [48, 49]. Two Sj22.6 IgE-binding epitopes showed sequence similarity with known IgE-binding epitopes from fish and grass pollen allergens [49]. Genome searches identified 13 *S. mansoni* tegumental-allergen-like proteins (TALs) that are differentially expressed throughout the parasite lifecycle [50]. In terms of allergenic activity, a luciferase-based test using a humanized rat basophilic leukemia cell line showed that SmTAL1 and SmTAL2 induce basophil activation [51].

Trematode “venom allergen-like proteins” (VALs) are similar to the wasp venom allergen Ves v 5. SmVAL6 is recognized by IgE from *S. mansoni*–infected individuals, with a higher IgE-binding frequency after anthelmintic treatment [52]. Besides, SmVAL4 induced IgE production in immunized BALB/c mice, generating allergic airway inflammation and passive cutaneous anaphylaxis, supporting the allergenic activity of this protein [53]. Other *S. mansoni* IgE-binding proteins are the IL-4-inducing principle from *S. mansoni* eggs (IPSE/alpha-1), a glycoprotein that induces IL-4 release from basophils [51, 54], and kappa-5, produced by the egg and miracidium stages, with a 45% IgE-binding frequency among patients infected with *S. mansoni* and 0% in uninfected controls [55].

In addition, *S. mansoni* ATP diphosphohydrolases (SmATPDases) also have allergenic properties [56], and some cysteine proteases of this parasite, such as cathepsin B1 (SmCB1), are similar to mite allergen Der p 1 [57, 58]. Furthermore, IgE from infected individuals recognizes antigens from schistosomula, including the extracellular vesicle-enriched larval extracellular vesicle protein 1 (SmLEV1) [59]. It is also important to note that *S. mansoni* has IgE-binding homologs to well-known allergens like tropomyosin, GSTs, profilin, lipocalin, cyclophilin, and phosphoglycerate kinase [52].

### *Toxocara *spp

There is experimental and epidemiological evidence that toxocariasis increases asthma susceptibility [60]. A meta-analysis revealed that children infected with *Toxocara spp.* are more likely to have asthma compared to non-infected children [61]. Human specific IgE antibodies detect several components in the ESPs of *T. canis* [62]; however, the identification of individual IgE-binding components from this parasite remains to be done.

### Hookworms

Most studies about these nematodes suggest that they are more associated with immunosuppression than with an increase in allergic symptoms [63], although some reports show positive associations between current or past hookworm infection and allergic rhinoconjunctivitis or wheezing [64, 65]. Hookworms can induce allergic inflammation in humans during their lifecycle, and the passage of larvae through the lungs can cause Löffler syndrome. In addition, *N. americanus* can induce basophil activation and degranulation in the absence of detectable specific IgE, probably due to early basophil sensitization or an effect mediated by hookworm-secreted proteases [66, 67]. Furthermore, larvae of animal hookworms, such as *Ancylostoma caninum*, can penetrate human skin and produce an intensely pruritic, erythematous, serpiginous eruption that is characterized histologically by an important eosinophilic infiltration, called larva migrans [68]. Although the presence of specific IgE against hookworm antigens has been demonstrated by serological assays, skin tests, and basophil histamine release, few of their IgE-binding components have been identified [69, 71]. One of these is the *N. americanus* calreticulin, which induces basophil histamine release and has little cross-reactivity with its human counterpart [72, 73].

Another hookworm IgE-binding molecule, *Na*-ASP-2, is abundantly secreted by *N. americanus* larvae upon entry into the host and is structurally similar to vespid venom allergens and SmVALs [74, 76]. *Na*-ASP-2 can elicit positive skin test results [77], supporting its allergenic activity. Both calreticulin and *Na*-ASP-2 have been studied as anti-hookworm vaccines [74, 78], but their allergenic properties have impeded these efforts. *Na*-ASP-2 induced generalized urticaria in three of seven individuals who received a single dose of a recombinant version of this protein, all of which had pre-existing *Na*-ASP-2-specific IgE [77]. Regarding *A. caninum*, Ac68 and Ac-ASP-1 are IgE-binding molecules from this parasite [79, 80].

### Filariae

Human filariasis has been associated with allergic manifestations and atopic sensitization. Microfilaria of *W. bancrofti* in the lungs can cause an allergic reaction that resembles asthma, a rare condition known as tropical pulmonary eosinophilia (TPE), which presents with cough, dyspnea, wheezing, chest pain, systemic manifestations (including fever and weight loss), peripheral blood eosinophilia > 3000/µL, and elevated total IgE > 1000 IU/mL. Patients with TPE have higher levels of filarial-specific IgE than those with other manifestations of filarial infection [81], and this condition can lead to irreversible pulmonary hypertension, pulmonary fibrosis, and chronic respiratory failure. Animal models of filariasis show that helminth-induced airway hyperresponsiveness is dependent on IL-4, IL-5, and eosinophil recruitment and degranulation, consistent with type 2 immune-mediated inflammation [82, 83].

Filariae induce a long-lasting IgE response and contain several IgE-binding components that are poorly characterized [84] such as the *O. volvulus* molecules Ov27, OvD5B, and OvGalBP [85, 86]. *W. bancrofti* and *B. malayi* components trigger histamine release from human basophils, whereas *B. malayi* antigens also induce positive skin test reactions [81, 84, 87]. Fourteen IgE-binding ESPs of *B. malayi* have been identified, including transthyretin-related protein (TTR), WbSXP-1, macrophage migration inhibitory factor, and gp15/400 [88•]. Interestingly, proteins belonging to the TTR family are the most dominant filarial antigens targeted by the human IgE response [88•]. Sensitization with TTR-binding IgE monoclonal antibodies induces anaphylaxis in mice upon challenge with TTR proteins [88•]. Bm23-25 is a *B. malayi* IgE-binding component with a potential role in the pathogenesis of TPE given that it is recognized by IgE present in the bronchoalveolar lavage fluid and serum of patients with this condition [89]. Bm23-25 is a homolog of mammalian γ-glutamyl transpeptidase (γ-GT) that induces a specific antibody response (including IgG1 and IgE) in BALB/c mice and exhibits cross-reactivity with human airway epithelium γ-GT [89, 90]. In addition, patients with TPE, but also healthy individuals from a filaria-endemic area, have elevated specific IgE levels against *B. malayi* γ-GT [91].

Many filarial proteins have homologs in allergenic sources such as mites and cockroaches, which can lead to cross-sensitization [92, 93]. For example, *B. tropicalis* trypsin (Blo t 3) shares amino acid sequence similarity with its *W. bancrofti* counterpart [93]. In addition, there is considerable amino acid sequence identity, 3D structure similarity, and cross-reactivity between mite and filarial tropomyosins (i.e., Der p 10 from *D. pteronyssinus* and OvTrop from *O. volvulus*), as well as between cockroach and filarial GSTs (i.e., Bla g 5 from *Blattella germanica* and WbGST from *W. bancrofti*) [94, 95]. Furthermore, vespid venom allergen homologs are present in *B. malayi* and *W. bancrofti* (e.g., Bm-VAL-1 and WbVAH) [96, 97].

## Helminth Allergens and Allergy Diagnosis

Since allergic responses and helminth immunity have several similarities, it is possible that some diagnostic parameters for allergy can be confounded by helminthiases. In rural areas of the tropics, severe helminthiases can diminish allergen skin testing results [98, 99], even in the presence of serum allergen-specific IgE [100]. In tropical urbanized settings, when using allergen extracts for skin testing or serodiagnosis, cross-reactivity between *A. lumbricoides* and HDM allergens (tropomyosin and GST) may confuse the diagnosis because sensitization to both organisms is very frequent [101]. In addition, there is evidence that this could also be important in some temperate zones [4, 102]. Fortunately, today it is possible to differentiate co-sensitization vs cross-reactivity between the two sources using molecular diagnosis [103]. Another constraint of serologic allergy diagnosis in patients with helminthiases is the presence of the carbohydrate epitope galactose-α1,3-galactose (α-Gal), which is expressed in non-primate mammalian proteins, such as the cat allergen Fel d 5 (cat IgA) and *A. lumbricoides* [104, 105].

**Fig. 3:**
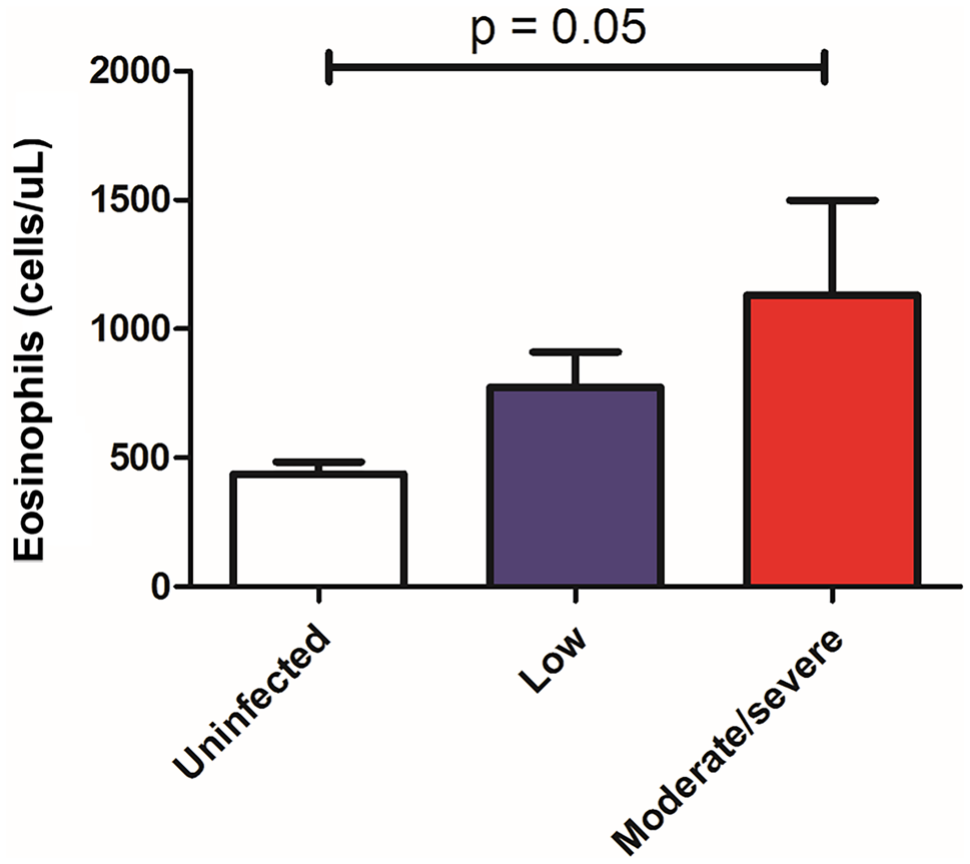
Blood eosinophil counts increase with the severity of *A. lumbricoides* infection. The number of eosinophils is expressed as absolute  cell counts/μL. Low infection < 2500 eggs/g. Moderate/Severe = equal or > 2500 eggs/g. A total of 82 subjects were included

Helminthiases could also limit the usefulness of type 2 asthma markers such as total IgE, fractional exhaled nitric oxide (FeNO), and blood eosinophils, which can impact decisions regarding the use of biologics for treatment. Both asthma and helminthiases run with high total IgE levels; in tropical zones, children reach high total IgE levels at the age of 3 years [106] (Fig. 2). Hence, specific IgE detection is a better criterion than total IgE for defining atopy in these locations. Blood eosinophilia has also been described in both asthmatic [107] and parasitized subjects [108]. Indeed, we found that eosinophil counts increased with the severity of *A. lumbricoides* infection [109] (Fig. 3). In addition, a comparison of asthmatic and non-asthmatic subjects living in an endemic tropical village, using a general linear model, showed that eosinophil counts were dependent on ascariasis rather than asthma (Zakzuk J, personal communication). Therefore, investigation of current helminth infection by stool examination can help define the origin of blood eosinophilia in asthmatic patients living in endemic zones of the tropics.

FeNO is an important tool for evaluating lung inflammation [110] and is currently used as a clinical biomarker of Th2 inflammation in asthma [111]. A previous study suggested that FeNO levels are higher in *A. lumbricoides* parasitized children in Ecuador [99]. However, in a cohort of adult asthmatic patients, we found no difference in FeNO levels between *Ascaris*-sensitized and non-sensitized patients [112]. There were also no differences in FeNO levels between children that were IgE-sensitized to the infection marker ABA-1. Since positive IgE reflects parasite exposure but not necessarily active infection, we also compared FeNO levels between parasitized and non-parasitized children (*n* = 99) from a rural village in Colombia. Preliminary unpublished results (Acevedo N, personal communication) showed no difference in FeNO levels between infected and non-infected children. Cases with lung migratory phase parasites, such as *A. lumbricoides* or *A. duodenalis*, had FeNO levels below 20 ppb (see some examples in Table 2). Therefore, although more research about this topic is needed, in tropical environments this important diagnostic tool should also be supported with patient’s stool examination.

**Table 2:** A descriptive of FeNO levels in children infected by nematodes with lung migratory phases in their lifecycle

**Subject ID**	**Age (years)**	**Nematode detected**	**Eggs per gram of feces**•	**FeNO level (ppb)**	**Blood eosinophil cell counts (eos/μL)** ••
LA012	7	*Ascaris lumbricoides*	72	16.5	1090
ST095	8	*Ascaris lumbricoides*	1824	19.5	600
ST093	9	*Ancylostoma duodenalis*	72	4	1930
ST094	7	*Ancylostoma duodenalis*	312	12	760
ST101	9	*Ancylostoma duodenalis*	120	18.5	1450

•As quantified by the Kato-Katz method

••Eosinophilia is considered above 500 eosinophils per microliter of blood

FeNO levels < 20 ppb are considered normal in children

*ppb*, parts per billion

## Conclusions

The allergenic activity of several helminth products (e.g., parasite extract, pseudocelomic fluid, and extracellular vesicles) has been demonstrated experimentally, although more research is needed to identify the molecules involved and the underlying mechanisms. Still, this information supports clinical and epidemiological evidence demonstrating the diverse pro-allergenic effects of helminthiases in humans, especially in areas where helminthiases are frequent. The modifications of the natural history of allergic diseases induced by helminth infections also impact their diagnosis and management.

## Data Availability

Acevedo N, personal communication.

## References

[1] Caraballo L (2018). The tropics, helminth infections and hygiene hypotheses. Expert Rev Clin Immunol.

[2] Caraballo L, Coronado S (2018). Parasite allergens. Mol Immunol.

[3] •• Arrais M, Maricoto T, Nwaru BI, Cooper PJ, Gama JMR, Brito M, et al. Helminth infections and allergic diseases: systematic review and meta-analysis of the global literature. J Allergy Clin Immunol. 2022;149(6):2139–52. **Important global analysis of the literature on the epidemiological evidence of helminth-allergy interactions.** 10.1016/j.jaci.2021.12.777 34968529

[4] • Jogi NO, Kitaba N, Storaas T, Schlunssen V, Triebner K, Holloway JW, et al. Ascaris exposure and its association with lung function, asthma, and DNA methylation in Northern Europe. J Allergy Clin Immunol. 2022;149(6):1960–9. **This work shows that helminthiases like ascariasis are still important in temperate regions.** 10.1016/j.jaci.2021.11.013 34996616

[5] • Cooper PJ, Figueiredo CA, Rodriguez A, Dos Santos LM, Ribeiro-Silva RC, Carneiro VL, et al. Understanding and controlling asthma in Latin America: a review of recent research informed by the SCAALA programme. Clinical and translational allergy. 2023;13(3):e12232. **Interesting discussion about the effects or urbanization and other socioeconomic factors on allergic diseases in Latin America.** 10.1002/clt2.12232 PMC10041090 36973960

[6] Acevedo N, Zakzuk J, Caraballo L (2019). House dust mite allergy under changing environments. Allergy Asthma Immunol Res.

[7] • Vacca F, Le Gros G. Tissue-specific immunity in helminth infections. Mucosal Immunol. 2022;15(6):1212–23. **This is an update of the basic mechanisms of helminth immunity from experiments in animals, focused in tissue-specific aspects.** 10.1038/s41385-022-00531-w PMC9178325 35680972

[8] Caraballo L, Valenta R, Puerta L, Pomes A, Zakzuk J, Fernandez-Caldas E, et al. The allergenic activity and clinical impact of individual IgE-antibody binding molecules from indoor allergen sources. World Allergy Organ J. 2020;13(5). 10.1016/j.waojou.2020.100118 PMC7195550 32373267

[9] Caraballo L, Zakzuk J, Acevedo N. Helminth-derived cystatins: the immunomodulatory properties of an Ascaris lumbricoides cystatin. Parasitology. 2021:1–13. 10.1017/S0031182021000214 33563346

[10] Ahumada V, Garcia E, Dennis R, Rojas MX, Rondon MA, Perez A (2015). IgE responses to Ascaris and mite tropomyosins are risk factors for asthma. Clin Exp Allergy.

[11] Caraballo L, Acevedo N, Zakzuk J (2019). Ascariasis as a model to study the helminth/allergy relationships. Parasite Immunol.

[12] Hunninghake GM, Soto-Quiros ME, Avila L, Ly NP, Liang C, Sylvia JS (2007). Sensitization to Ascaris lumbricoides and severity of childhood asthma in Costa Rica. J Allergy Clin Immunol.

[13] Buendia E, Zakzuk J, Mercado D, Alvarez A, Caraballo L. The IgE response to Ascaris molecular components is associated with clinical indicators of asthma severity. World Allergy Organ J. 2015;8(1):8. 10.1186/s40413-015-0058-z PMC4347909 25780492

[14] Lee TD, Xie CY (1995). IgE regulation by nematodes: the body fluid of Ascaris contains a B-cell mitogen. J Allergy Clin Immunol.

[15] Caraballo LAN, Buendia E (2015). Human ascariasis increases the allergic response and allergic symptoms. Curr Trop Med Rep.

[16] Acevedo N, Sanchez J, Zakzuk J, Bornacelly A, Quiroz C, Alvarez A (2012). Particular characteristics of allergic symptoms in tropical environments: follow up to 24 months in the FRAAT birth cohort study. BMC Pulm Med.

[17] Zakzuk J, Bornacelly A, Mercado D, Sánchez J, Acevedo N, Caraballo L. The evolution of IgE sensitization to Ascaris allergenic components in early infancy. In: Galli SJ, You-Young Kim, editor. Allergic diseases: from mechanisms to cure. Italy: Pacini Editore. 2014:29–31.

[18] Acevedo N, Bornacelly A, Mercado D, Unneberg P, Mittermann I, Valenta R (2016). Genetic variants in CHIA and CHI3L1 are associated with the IgE response to the Ascaris resistance marker ABA-1 and the birch pollen allergen Bet v 1. PLoS ONE.

[19] • Zakzuk J, Acevedo N, Harb H, Eick L, Renz H, Potaczek DP, et al. IgE levels to Ascaris and house dust mite allergens are associated with increased histone acetylation at key type-2 immune genes. Front Immunol. 2020;11:756. **This study shows epigenetic mechanisms underlying the boosting of specific IgE against house dust mite allergens during human ascariasis.** 10.3389/fimmu.2020.00756 PMC7204827 32425942

[20] Acevedo N, Sanchez J, Erler A, Mercado D, Briza P, Kennedy M (2009). IgE cross-reactivity between Ascaris and domestic mite allergens: the role of tropomyosin and the nematode polyprotein ABA-1. Allergy.

[21] Acevedo NEA, Briza P, Puccio F, Ferreira F, Caraballo L (2011). Allergenicity of Ascaris lumbricoides tropomyosin and IgE sensitization among asthmatic patients in a tropical environment. Int Arch of Allergy Immunol.

[22] Acevedo N, Mohr J, Zakzuk J, Samonig M, Briza P, Erler A (2013). Proteomic and immunochemical characterization of glutathione transferase as a new allergen of the nematode Ascaris lumbricoides. PLoS ONE.

[23] •• Ahumada V, Manotas M, Zakzuk J, Aglas L, Coronado S, Briza P, et al. Identification and physicochemical characterization of a new allergen from Ascaris lumbricoides. Int J Mol Sci. 2020;21(24). **This article describes the last obtained *****A. lumbricoides***** allergen, including several technical procedures for supporting its allergenic activity.** 10.3390/ijms21249761 PMC7767342 33371317

[24] Ahumada V, Zakzuk J, Coronado S, Aglaz L, Araujo G, Briza P, et al. Identification of a new, no cross-reacting allergen from Ascaris lumbricoides [abstract]. World Allergy Organ J. 2020;13(8).

[25] Acevedo N, Erler A, Briza P, Puccio F, Ferreira F, Caraballo L (2011). Allergenicity of Ascaris lumbricoides tropomyosin and IgE sensitization among asthmatic patients in a tropical environment. Int Arch Allergy Immunol.

[26] Caraballo L, Lopata AL, Acevedo N. Tropomyosins. In: Hoffmann-Sommergruber K, Hilger C, Santos A, de las Vecillas L, Dramburg S, editors. Molecular allergology user’s guide. 2nd ed. Zurich: European Academy of Allergy and Clinical Immunology; 2022.

[27] Mueller GA, Pedersen LC, Glesner J, Edwards LL, Zakzuk J, London RE (2015). Analysis of glutathione S-transferase allergen cross-reactivity in a North American population: relevance for molecular diagnosis. J Allergy Clin Immunol.

[28] • Zakzuk J, Lozano A, Caraballo L. Allergological importance of invertebrate glutathione transferases in tropical environments. Front Allergy. 2021;2:695262. **A comprehensive review of GSTs from different organisms and their impact on allergic diseases.** 10.3389/falgy.2021.695262 PMC8974725 35387058

[29] McSharry C, Xia Y, Holland CV, Kennedy MW (1999). Natural immunity to Ascaris lumbricoides associated with immunoglobulin E antibody to ABA-1 allergen and inflammation indicators in children. Infect Immun.

[30] Rasul TF, Bergholz DR, Faiz A (2021). Latent Strongyloides stercoralis in an asymptomatic male with chronic peripheral eosinophilia. Cureus.

[31] Araujo ES, de Jesus Pereira CA, de Moura Pereira AT, Moreira JM, de Rezende MC, Rodrigues JL (2016). The role of IL-33/ST2, IL-4, and eosinophils on the airway hyperresponsiveness induced by Strongyloides venezuelensis in BALB/c mice. Parasitol Res.

[32] de las Marinas MD, Martorell A, Felix R, Cerdá JC, García A, Navalpotro D. Strongyloidiasis: an emerging infectious disease that simulates allergic diseases. J Investig Allergol Clin Immunol. 2012;22(4):286–7. 22812198

[33] • Hazan G, Orscheln RC, Kertz L, Rivera-Spoljaric K. A child with chronic cough and eosinophilia secondary to Strongyloides stercoralis infection. Pediatr Pulmonol. 2022;57(10):2562–4. **This is an example of what is still happening during helminth infections.** 10.1002/ppul.26048 35778783

[34] Salam R, Sharaan A, Jackson SM, Solis RA, Zuberi J (2020). Strongyloides hyperinfection syndrome: a curious case of asthma worsened by systemic corticosteroids. Am J Case Rep.

[35] Tamarozzi F, Martello E, Giorli G, Fittipaldo A, Staffolani S, Montresor A (2019). Morbidity associated with chronic Strongyloides stercoralis infection: a systematic review and meta-analysis. Am J Trop Med Hyg.

[36] Neva FA, Gam AA, Maxwell C, Pelletier LL (2001). Skin test antigens for immediate hypersensitivity prepared from infective larvae of Strongyloides stercoralis. Am J Trop Med Hyg.

[37] Ravi V, Ramachandran S, Thompson RW, Andersen JF, Neva FA (2002). Characterization of a recombinant immunodiagnostic antigen (NIE) from Strongyloides stercoralis L3-stage larvae. Mol Biochem Parasitol.

[38] Ravi V, Nutman TB, Andersen JF, Neva FA, King TP (2005). Strongyloides stercoralis recombinant NIE antigen shares epitope with recombinant Ves v 5 and Pol a 5 allergens of insects. Am J Trop Med Hyg.

[39] Varatharajalu R, Parandaman V, Ndao M, Andersen JF, Neva FA (2011). Strongyloides stercoralis excretory/secretory protein strongylastacin specifically recognized by IgE antibodies in infected human sera. Microbiol Immunol.

[40] Ahmad H, Arifin N, Nolan TJ, Lok JB, Anuar NS, Noordin R. Strongyloides-specific IgE phage cDNA clones and development of a novel ELISA for strongyloidiasis. Diagnostics (Basel). 2021;11(6). 10.3390/diagnostics11060985 PMC8228214 34071716

[41] Medeiros M, Figueiredo JP, Almeida MC, Matos MA, Araujo MI, Cruz AA (2003). Schistosoma mansoni infection is associated with a reduced course of asthma. J Allergy Clin Immunol.

[42] van den Biggelaar AH, van Ree R, Rodrigues LC, Lell B, Deelder AM, Kremsner PG (2000). Decreased atopy in children infected with Schistosoma haematobium: a role for parasite-induced interleukin-10. Lancet.

[43] Smits HH, Hammad H, van Nimwegen M, Soullie T, Willart MA, Lievers E (2007). Protective effect of Schistosoma mansoni infection on allergic airway inflammation depends on the intensity and chronicity of infection. J Allergy Clin Immunol.

[44] Webb EL, Nampijja M, Kaweesa J, Kizindo R, Namutebi M, Nakazibwe E (2016). Helminths are positively associated with atopy and wheeze in Ugandan fishing communities: results from a cross-sectional survey. Allergy.

[45] Nkurunungi G, Kabagenyi J, Nampijja M, Sanya RE, Walusimbi B, Nassuuna J, et al. Schistosoma mansoni-specific immune responses and allergy in Uganda. Parasite Immunol. 2018;40(1). 10.1111/pim.12506 PMC5767746 29171863

[46] Yombo DJK, Mentink-Kane MM, Wilson MS, Wynn TA, Madala SK (2019). Heat shock protein 70 is a positive regulator of airway inflammation and goblet cell hyperplasia in a mouse model of allergic airway inflammation. J Biol Chem.

[47] Dunne DW, Webster M, Smith P, Langley JG, Richardson BA, Fulford AJ (1997). The isolation of a 22 kDa band after SDS-PAGE of Schistosoma mansoni adult worms and its use to demonstrate that IgE responses against the antigen(s) it contains are associated with human resistance to reinfection. Parasite Immunol.

[48] Fitzsimmons CM, Stewart TJ, Hoffmann KF, Grogan JL, Yazdanbakhsh M, Dunne DW. Human IgE response to the Schistosoma haematobium 22.6 kDa antigen. Parasite Immunol. 2004;26(8–9):371–6. 10.1111/j.0141-9838.2004.00721.x 15679635

[49] Santiago ML, Hafalla JC, Kurtis JD, Aligui GL, Wiest PM, Olveda RM, et al. Identification of the Schistosoma japonicum 22.6-kDa antigen as a major target of the human IgE response: similarity of IgE-binding epitopes to allergen peptides. Int Arch Allergy Immunol. 1998;117(2):94–104. 10.1159/000023995 9784652

[50] Fitzsimmons CM, Jones FM, Stearn A, Chalmers IW, Hoffmann KF, Wawrzyniak J (2012). The Schistosoma mansoni tegumental-allergen-like (TAL) protein family: influence of developmental expression on human IgE responses. PLoS Negl Trop Dis.

[51] Wan D, Ludolf F, Alanine DG, Stretton O, Ali Ali E, Al-Barwary N (2014). Use of humanised rat basophilic leukaemia cell line RS-ATL8 for the assessment of allergenicity of Schistosoma mansoni proteins. PLoS Negl Trop Dis.

[52] Farnell EJ, Tyagi N, Ryan S, Chalmers IW, Pinot de Moira A, Jones FM, et al. Known allergen structures predict Schistosoma mansoni IgE-binding antigens in human infection. Front Immunol. 2015;6:26. 10.3389/fimmu.2015.00026 PMC4315118 25691884

[53] Farias LP, Rodrigues D, Cunna V, Rofatto HK, Faquim-Mauro EL, Leite LC (2012). Schistosoma mansoni venom allergen like proteins present differential allergic responses in a murine model of airway inflammation. PLoS Negl Trop Dis.

[54] Meyer NH, Mayerhofer H, Tripsianes K, Blindow S, Barths D, Mewes A (2015). A crystallin fold in the interleukin-4-inducing principle of Schistosoma mansoni eggs (IPSE/alpha-1) mediates IgE binding for antigen-independent basophil activation. J Biol Chem.

[55] Schramm G, Hamilton JV, Balog CI, Wuhrer M, Gronow A, Beckmann S (2009). Molecular characterisation of kappa-5, a major antigenic glycoprotein from Schistosoma mansoni eggs. Mol Biochem Parasitol.

[56] Braga Emidio N, Antonia do Nascimento Gusmao M, Castro Borges W, Ryuichi Nakaie C, Gomes Vasconcelos E, Faria Pinto P. Identification of a linear IgE inducing epitope on the SmATPDase1 surface. Acta Biochim Biophys Sin (Shanghai). 2017;49(6):564–6. 10.1093/abbs/gmx031 28398461

[57] Furmonaviciene R, Sewell HF, Shakib F (2000). Comparative molecular modelling identifies a common putative IgE epitope on cysteine protease allergens of diverse sources. Clin Exp Allergy.

[58] de Oliveira Fraga LA, Lamb EW, Moreno EC, Chatterjee M, Dvorak J, Delcroix M (2010). Rapid induction of IgE responses to a worm cysteine protease during murine pre-patent schistosome infection. BMC Immunol.

[59] Gasan TA, Kuipers ME, Roberts GH, Padalino G, Forde-Thomas JE, Wilson S (2021). Schistosoma mansoni larval extracellular vesicle protein 1 (SmLEV1) is an immunogenic antigen found in EVs released from pre-acetabular glands of invading cercariae. PLoS Negl Trop Dis.

[60] Mendonca LR, Veiga RV, Dattoli VC, Figueiredo CA, Fiaccone R, Santos J (2012). Toxocara seropositivity, atopy and wheezing in children living in poor neighbourhoods in urban Latin American. PLoS Negl Trop Dis.

[61] Aghaei S, Riahi SM, Rostami A, Mohammadzadeh I, Javanian M, Tohidi E, et al. Toxocara spp. infection and risk of childhood asthma: a systematic review and meta-analysis. Acta Trop. 2018;182:298–304. 10.1016/j.actatropica.2018.03.022 29573999

[62] Rubinsky-Elefant G, Hoshino-Shimizu S, Jacob CM, Sanchez MC, Ferreira AW (2011). Potential immunological markers for diagnosis and therapeutic assessment of toxocariasis. Rev Inst Med Trop Sao Paulo.

[63] Chapman PR, Giacomin P, Loukas A, McCarthy JS (2021). Experimental human hookworm infection: a narrative historical review. PLoS Negl Trop Dis.

[64] Wordemann M, Diaz RJ, Heredia LM, Collado Madurga AM, Ruiz Espinosa A, Prado RC (2008). Association of atopy, asthma, allergic rhinoconjunctivitis, atopic dermatitis and intestinal helminth infections in Cuban children. Trop Med Int Health.

[65] Sangsupawanich P, Mahakittikun V, Chongsuvivatwong V, Mo-suwan L, Choprapawon C (2010). Effect of helminthic infections together with mite allergen exposure on the risk of wheeze in preschool children. Asian Pac J Allergy Immunol.

[66] Falcone FH, Telford G, Hooi D, Brown AP, Seabra R, Feary J, et al. Antigen-driven basophil activation is indicative of early Necator americanus infection in IgE-seronegative patients. J Allergy Clin Immunol. 2009;124(6):1343–50 e7. 10.1016/j.jaci.2009.07.039 19800679

[67] Phillips C, Coward WR, Pritchard DI, Hewitt CR (2003). Basophils express a type 2 cytokine profile on exposure to proteases from helminths and house dust mites. J Leukoc Biol.

[68] Balfour E, Zalka A, Lazova R (2002). Cutaneous larva migrans with parts of the larva in the epidermis. Cutis.

[69] Griffiths GD, Hornby RJ, Scott L, Pritchard DI, Brown AP, Hooi DSW (2008). Development of a model of hookworm infection exhibiting salient characteristics of human infection. Am J Trop Med Hyg.

[70] de Hurtado I, Layrisse M (1968). Epidemiologic role of skin hypersensitivity in hookworm disease. Am J Trop Med Hyg.

[71] Loukas A, Croese J, Opdebeeck J, Prociv P (1992). Detection of antibodies to secretions of Ancylostoma caninum in human eosinophilic enteritis. Trans R Soc Trop Med Hyg.

[72] Pritchard DI, Brown A, Kasper G, McElroy P, Loukas A, Hewitt C (1999). A hookworm allergen which strongly resembles calreticulin. Parasite Immunol.

[73] Pritchard DI, Quinnell RJ, Brown A, Bockarie MJ, Caddick R, Hooi DSW (2007). Basophil competence during hookworm (Necator americanus) infection. Am J Trop Med Hyg.

[74] Bethony J, Loukas A, Smout M, Brooker S, Mendez S, Plieskatt J (2005). Antibodies against a secreted protein from hookworm larvae reduce the intensity of hookworm infection in humans and vaccinated laboratory animals. FASEB J.

[75] Asojo OA (2011). Structure of a two-CAP-domain protein from the human hookworm parasite Necator americanus. Acta Crystallogr D Biol Crystallogr.

[76] Kelleher A, Darwiche R, Rezende WC, Farias LP, Leite LC, Schneiter R (2014). Schistosoma mansoni venom allergen-like protein 4 (SmVAL4) is a novel lipid-binding SCP/TAPS protein that lacks the prototypical CAP motifs. Acta Crystallogr D Biol Crystallogr.

[77] Diemert DJ, Pinto AG, Freire J, Jariwala A, Santiago H, Hamilton RG, et al. Generalized urticaria induced by the Na-ASP-2 hookworm vaccine: implications for the development of vaccines against helminths. J Allergy Clin Immunol. 2012;130(1):169–76 e6. 10.1016/j.jaci.2012.04.027 22633322

[78] Winter JA, Davies OR, Brown AP, Garnett MC, Stolnik S, Pritchard DI (2005). The assessment of hookworm calreticulin as a potential vaccine for necatoriasis. Parasite Immunol.

[79] Ghosh K, Hotez PJ (1999). Antibody-dependent reductions in mouse hookworm burden after vaccination with Ancylostoma caninum secreted protein 1. J Infect Dis.

[80] Loukas A, Opdebeeck J, Croese J, Prociv P (1994). Immunologic incrimination of Ancylostoma caninum as a human enteric pathogen. Am J Trop Med Hyg.

[81] Ottesen EA, Neva FA, Paranjape RS, Tripathy SP, Thiruvengadam KV, Beaven MA (1979). Specific allergic sensitisation to filarial antigens in tropical eosinophilia syndrome. Lancet.

[82] Cadman ET, Thysse KA, Bearder S, Cheung AY, Johnston AC, Lee JJ (2014). Eosinophils are important for protection, immunoregulation and pathology during infection with nematode microfilariae. PLoS Pathog.

[83] Mehlotra RK, Hall LR, Haxhiu MA, Pearlman E (2001). Reciprocal immunomodulatory effects of gamma interferon and interleukin-4 on filaria-induced airway hyperresponsiveness. Infect Immun.

[84] Mitre E, Nutman TB (2006). IgE memory: persistence of antigen-specific IgE responses years after treatment of human filarial infections. J Allergy Clin Immunol.

[85] Garraud O, Nkenfou C, Bradley JE, Perler FB, Nutman TB (1995). Identification of recombinant filarial proteins capable of inducing polyclonal and antigen-specific IgE and IgG4 antibodies. J Immunol.

[86] Klion AD, Donelson JE (1994). OvGalBP, a filarial antigen with homology to vertebrate galactoside-binding proteins. Mol Biochem Parasitol.

[87] Grove DI, Cabrera BD, Valeza FS, Guinto RS, Ash LR, Warren KS (1977). Sensitivity and specificity of skin reactivity to Brugia malayi and Dirofilaria immitis antigens in Bancroftian and Malayan filariasis in the Philippines. Am J Trop Med Hyg.

[88] • Hadadianpour A, Daniel J, Zhang J, Spiller BW, Makaraviciute A, DeWitt AM, et al. Human IgE mAbs identify major antigens of parasitic worm infection. J Allergy Clin Immunol. 2022;150(6):1525–33. **This study describes several IgE-binding ESPs using MoAbs showing that TTR are the main inducers of IgE response against filaria and have allergenic activity inducing anaphylaxis in mice.** 10.1016/j.jaci.2022.05.022 PMC9742163 35760390

[89] Lobos E, Zahn R, Weiss N, Nutman TB (1996). A major allergen of lymphatic filarial nematodes is a parasite homolog of the γ-glutamyl transpeptidase. Mol Med.

[90] Gounni AS, Spanel-Borowski K, Palacios M, Heusser C, Moncada S, Lobos E (2001). Pulmonary inflammation induced by a recombinant Brugia malayi γ-glutamyl transpeptidase homolog: involvement of humoral autoimmune responses. Mol Med.

[91] Lobos E, Nutman TB, Hothersall JS, Moncada S (2003). Elevated immunoglobulin E against recombinant Brugia malayi gamma-glutamyl transpeptidase in patients with Bancroftian filariasis: association with tropical pulmonary eosinophilia or putative immunity. Infect Immun.

[92] Santiago Hda C, Ribeiro-Gomes FL, Bennuru S, Nutman TB (2015). Helminth infection alters IgE responses to allergens structurally related to parasite proteins. J Immunol.

[93] Bielory BP, Mainardi T, Rottem M (2013). Evolutionary immune response to conserved domains in parasites and aeroallergens. Allergy Asthma Proc.

[94] Santiago HC, Bennuru S, Boyd A, Eberhard M, Nutman TB (2011). Structural and immunologic cross-reactivity among filarial and mite tropomyosin: implications for the hygiene hypothesis. J Allergy Clin Immunol.

[95] Santiago HC, LeeVan E, Bennuru S, Ribeiro-Gomes F, Mueller E, Wilson M, et al. Molecular mimicry between cockroach and helminth glutathione S-transferases promotes cross-reactivity and cross-sensitization. J Allergy Clin Immunol. 2012;130(1):248–56 e9. 10.1016/j.jaci.2012.02.045 PMC3387355 22541242

[96] Darwiche R, Lugo F, Drurey C, Varossieau K, Smant G, Wilbers RHP (2018). Crystal structure of Brugia malayi venom allergen-like protein-1 (BmVAL-1), a vaccine candidate for lymphatic filariasis. Int J Parasitol.

[97] Anand SB, Gnanasekar M, Thangadurai M, Prabhu PR, Kaliraj P, Ramaswamy K (2007). Immune response studies with Wuchereria bancrofti vespid allergen homologue (WbVAH) in human lymphatic filariasis. Parasitol Res.

[98] Cooper PJ, Chico ME, Rodrigues LC, Ordonez M, Strachan D, Griffin GE (2003). Reduced risk of atopy among school-age children infected with geohelminth parasites in a rural area of the tropics. J Allergy Clin Immunol.

[99] Cooper PJ, Chis Ster I, Chico ME, Vaca M, Oviedo Y, Maldonado A (2021). Impact of early life geohelminths on wheeze, asthma and atopy in Ecuadorian children at 8 years. Allergy.

[100] Zakzuk J, Casadiego S, Mercado A, Alvis-Guzman N, Caraballo L (2018). Ascaris lumbricoides infection induces both, reduction and increase of asthma symptoms in a rural community. Acta Trop.

[101] Acevedo N, Caraballo L (2011). IgE cross-reactivity between Ascaris lumbricoides and mite allergens: possible influences on allergic sensitization and asthma. Parasite Immunol.

[102] Ghahremani GG, Hahn ME (2022). Resurgence of intestinal ascariasis among adults: radiological diagnosis and clinical implications. Abdom Radiol (NY).

[103] Dramburg S, Hilger C, Santos AF, de Las Vecillas L, Aalberse RC, Acevedo N, et al. EAACI Molecular Allergology User's Guide 2.0. Pediatr Allergy Immunol. 2023 Mar;34 Suppl 28:e13854. 10.1111/pai.13854 37186333

[104] •• Murangi T, Prakash P, Moreira BP, Basera W, Botha M, Cunningham S, et al. Ascaris lumbricoides and ticks associated with sensitization to galactose alpha1,3-galactose and elicitation of the alpha-gal syndrome. J Allergy Clin Immunol. 2022;149(2):698–707 e3. **In this important work, the existence of alpha-gal in *****A. lumbricoides***** and other sources is demonstrated by several approaches.** 10.1016/j.jaci.2021.07.018 34333031

[105] Arkestal K, Sibanda E, Thors C, Troye-Blomberg M, Mduluza T, Valenta R (2011). Impaired allergy diagnostics among parasite-infected patients caused by IgE antibodies to the carbohydrate epitope galactose-alpha 1,3-galactose. J Allergy Clin Immunol.

[106] Zakzuk J, Acevedo N, Cifuentes L, Bornacelly A, Sanchez J, Ahumada V (2013). Early life IgE responses in children living in the tropics: a prospective analysis. Pediatr Allergy Immunol.

[107] Price DB, Rigazio A, Campbell JD, Bleecker ER, Corrigan CJ, Thomas M (2015). Blood eosinophil count and prospective annual asthma disease burden: a UK cohort study. Lancet Respir Med.

[108] Yanola J, Nachaiwieng W, Duangmano S, Prasannarong M, Somboon P, Pornprasert S (2018). Current prevalence of intestinal parasitic infections and their impact on hematological and nutritional status among Karen hill tribe children in Omkoi District, Chiang Mai Province. Thailand Acta Trop.

[109] Peñaranda Garcia D. Effects of Ascaris lumbricoides in eosinophils, regulatory B cells and asthma severity in asthmatic patients from a helminth endemic populatio [Master's Thesis]. Cartagena, Colombia: Institute for Immunological Research, University of Cartagena; 2019.

[110] Escamilla-Gil JM, Fernandez-Nieto M, Acevedo N (2022). Understanding the cellular sources of the fractional exhaled nitric oxide (FeNO) and its role as a biomarker of type 2 inflammation in asthma. Biomed Res Int.

[111] Porsbjerg C, Melen E, Lehtimaki L, Shaw D (2023). Asthma Lancet.

[112] De Vivero MM, Escamilla JM, Espinoza H, Regino R, Florez de Arco L, Caraballo L, et al. Type 2 inflammation biomarkers in adult asthmatic patients from a tropical environment and IgE sensitized to the helminth Ascaris [abstract]. Allergy. 2021;76(S110):424–582.

